# Photocatalytic degradation of Metronidazole with illuminated TiO_2_ nanoparticles

**DOI:** 10.1186/s40201-015-0194-y

**Published:** 2015-04-21

**Authors:** Mahdi Farzadkia, Edris Bazrafshan, Ali Esrafili, Jae-Kyu Yang, Mehdi Shirzad-Siboni

**Affiliations:** Department of Environmental Health Engineering, School of public Health, Iran University of Medical Sciences, Tehran, Iran; Health Promotion Research Center, Zahedan University of Medical Sciences, Zahedan, Iran; Divisions of General Education, Kwangwoon University, Seoul, 139-701 South Korea

**Keywords:** Titanium dioxide, Metronidazole, Photocatalysis, Kinetics

## Abstract

Metronidazole (MNZ) is a brand of nitroimidazole antibiotic, which is generally used in clinical applications and extensively used for the treatment of infectious diseases caused by anaerobic bacteria and protozoans. The aim of this investigation was to degrade MNZ with illuminated TiO_2_ nanoparticles at different catalyst dosage, contact time, pH, initial MNZ concentration and lamp intensity. Maximum removal of MNZ was observed at near neutral pH. Removal efficiency was decreased by increasing dosage and initial MNZ concentration. The reaction rate constant (*k*_*obs*_) was decreased from 0.0513 to 0.0072 min^−1^ and the value of electrical energy per order (E_Eo_) was increased from 93.57 to 666.67 (kWh/m^3^) with increasing initial MNZ concentration from 40 to 120 mg/L, respectively. The biodegradability estimated from the BOD5/COD ratio was increased from 0 to 0.098. The photocatalyst demonstrated proper photocatalytic activity even after five successive cycles. Finally, UV/TiO_2_ is identified as a promising technique for the removal of antibiotic with high efficiency in a relatively short reaction time.

## Background

Recently, several different types of emerging contaminants in water systems are known as new environmental hazards those need to be treated with suitable methods [1]. As various pharmaceutical compounds have been used since the 1950s due to rapid population growth and development of medical science, several pharmaceutical compounds have been found in surface water, ground water and effluents from wastewater treatment plants. Metronidazole (2-methyl-5-nitroimidazole-1-ethanol) has been widely used to treat infections caused by anaerobic bacteria, bacteroides and protozoa [2-4]. Residual concentrations of metronidazole (MNZ) in surface waters and wastewater are 1 ~ 10 ng/L [5,6]. As MNZ is non-biodegradable and highly soluble in water, it can be accumulated in the aquatic environment [7,8]. Elimination of MNZ from water system is an important issue considering its toxicity, potential mutagenicity and carcinogenity [7,8]. In order to remove MNZ, many techniques such as adsorption [9,10], reduction with nanoscale zero-valent iron particles [11], biological methods [12,13], ozonation technology [14], photolysis [15], Fenton and photo-Fenton processes [16], heterogeneous photocatalysis [15,17,18] and electro-Fenton process with a Ce/SnO_2_–Sb coated titanium anode [1] have been applied.

Adsorption is widely used method for the treatment of wastewater containing toxic organic compounds. However, it just transfer contaminants from water to a solid phase without any degradation [9,10,19,20]. Biological method is also known as one of the suggested techniques. However this method generally requires long periods for treatment [12,21]. Oxidation is a promising process but sometimes it is regarded as a limited process due to the formation of intermediates with higher toxicity than the parent compound [5,8,22]. Therefore near complete mineralization of MNZ is the most relevant option. For this purpose, advanced oxidation process (AOP) is regarded as a promising option to treat wastewater containing MNZ due to a complete mineralization of parent material as well as lack of selectivity [7,23]. Generally AOPs involve generation of hydroxyl radicals through UV/photocatalyst, UV/H_2_O_2_ and UV/O_3_ processes [24-26]. Among these methods, photocatalytic reaction using TiO_2_/UV can treat non-biodegradable organic compounds to biodegradable species [23,24,27]. Considering characteristics of the AOP, it can be used as pre- or post-treatment process in wastewater treatment because of its installation easiness in conventional wastewater treatment facilities [23,24,27].

Therefore, in the present work, P-25 TiO_2_ was selected as a catalyst in the photocatalytic removal of MNZ. Effects of several operational parameters including pH, TiO_2_ dosage and MNZ concentration on photocatalytic degradation of MNZ were investigated. Kinetic parameters for the photocatalytic degradation were obtained by application of the Langmuir–Hinshelwood (L–H) model. Finally, electrical energy per order (E_Eo_) was obtained to evaluate cost-efficiency of the processes used in this research.

## Material and methods

### Chemicals

Analytical grade of MNZ (C_6_H_9_N_3_O_3_; 99% chemical reagent) was purchased from Merck (Darmesdat, Germany) and its physical and chemical characteristics are summarized in Table 1. Potassium dihydrogen phosphate (KH_2_PO_4_) and acetonitrile (99.7%, HPLC grade) were purchased from Merck. P-25 TiO_2_ (80/20 mixture of anatase and rutile) was obtained from Degussa Corp. It has approximately spherical shape and has greater than 99.5% purity. The specific surface area of the TiO_2_ particles was 50 ± 15 m^2^/g according to Evonik-Industrial Co. The average size of the TiO_2_ particles was 21 nm. The antibiotic aqueous solution was prepared by dissolving 1 g of MNZ in 1 L distilled water. The antibiotic aqueous solution was prepared weekly and stored at 4°C. Initial COD and BOD_5_/COD ratio of 1000 mg/L MNZ was 126 mg/L and approximately 0, respectively.

**Table 1:** **Physical and chemical properties of MNZ**

**Characteristic**	**Metronidazole antibiotic (MNZ)**
Molecular structure	
Molecular formula	C_6_H_9_N_3_O_3_
Molecular weight (g/mol)	171.2
Water solubility (g/L)	9.5
pK_a_	2.55
Melting point (°C)	159-163
K_H_ (mol/dm^3^.atm)	5.92 × 10^7^
V_p_ (Pa)	4.07 × 10^−7^

**Figure 1:**
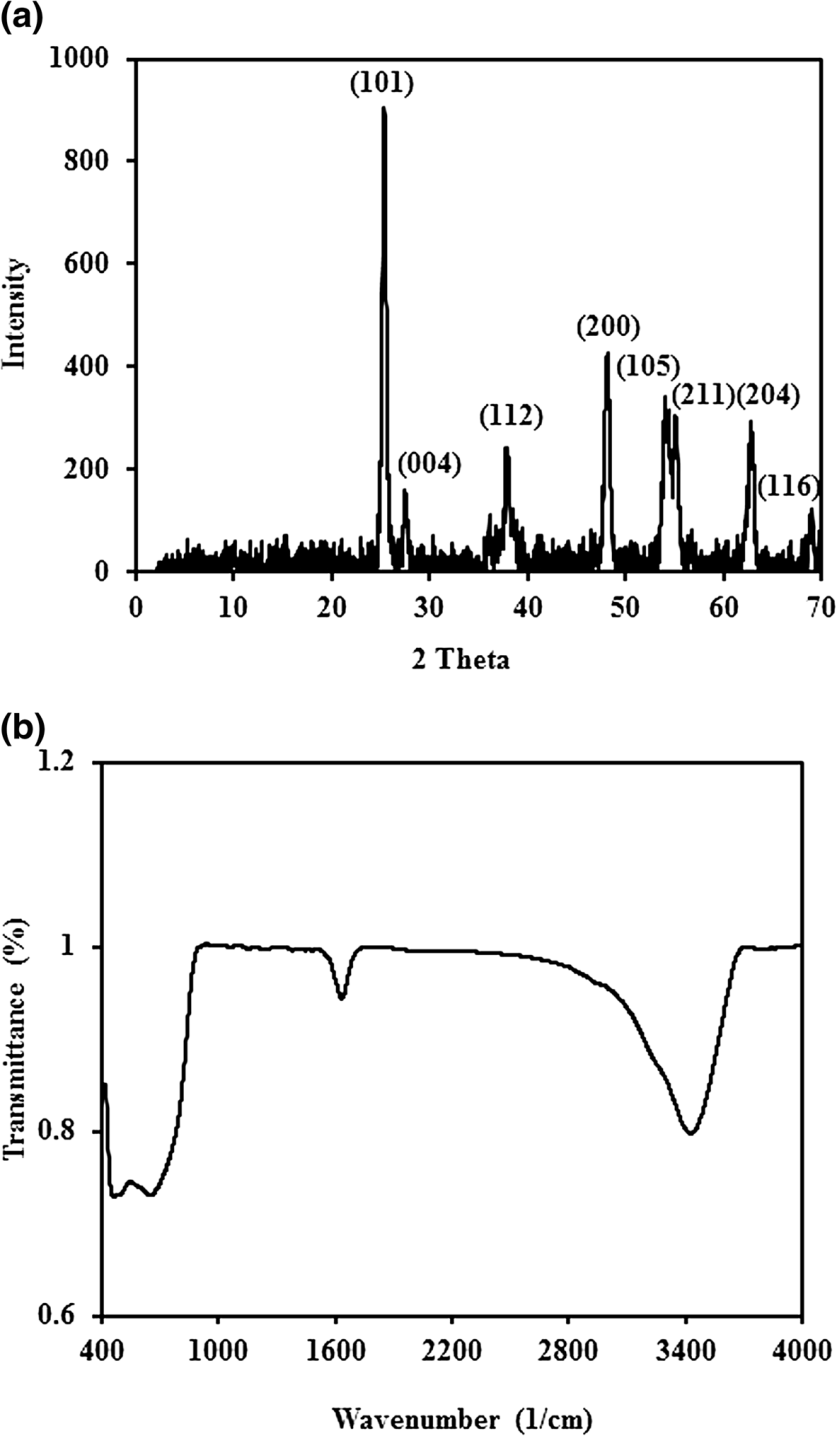
Characteristics of titanium dioxide: **(a)** XRD pattern **(b)** FT-IR pattern.

Figure 1a and b shows X-ray diffraction (XRD) and Fourier transform infrared spectroscopy (FT-IR) image of TiO_2_, respectively. The main peaks at 2θ values of 25.367, 37.909, 38.667, 48.158, 54.051, 55.204, 62.817 and 68.976 were correspond to the (101), (004), (112), (200), (105), (211), (204) and (116) planes of P-25 TiO_2_ (JCPDS card no. 36–1451). FT-IR analysis of TiO_2_ was performed in the range of 400–4000 1/cm (Figure 1b). The absorption bands at 438 1/cm and 620 1/cm was attributed to the E_g_ and A_2g_ mode, respectively. The pH_ZPC_ of TiO_2_ nanocatalyst was determined adopting the previously reported method.

### Experimental set-up

**Figure 2:**
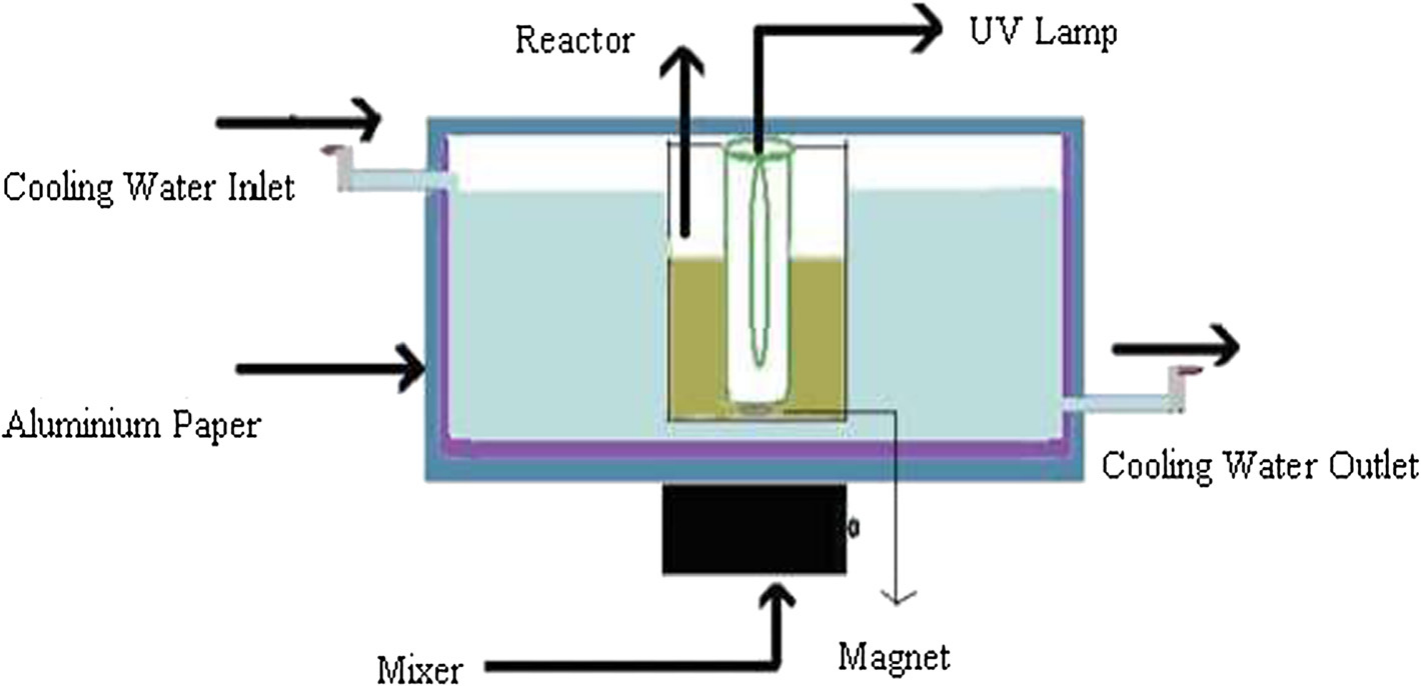
A schematic diagram of the experimental reactor.

The experimental reactor used for the photocatalytic degradation of MNZ is shown in Figure 2. The total volume of the reactor was 2 L with working volume of 1 L. The solution in the reactor was constantly stirred via a magnetic stirrer (170 rpm). A 125 W medium-pressure UVC lamp emitting maximum wavelength at 247.3 nm and a low-pressure UV lamp with irradiation intensity 8 W were applied as light sources. The light intensity of the UVC lamp was equal to 1020 μw/cm^2^ measured by a Spectroline model DRC-100X digital radiometer combined with a DIX-365 radiation sensor (ShokofanTosee, Company, Iran).

### Experimental procedure and analysis

**Figure 3:**
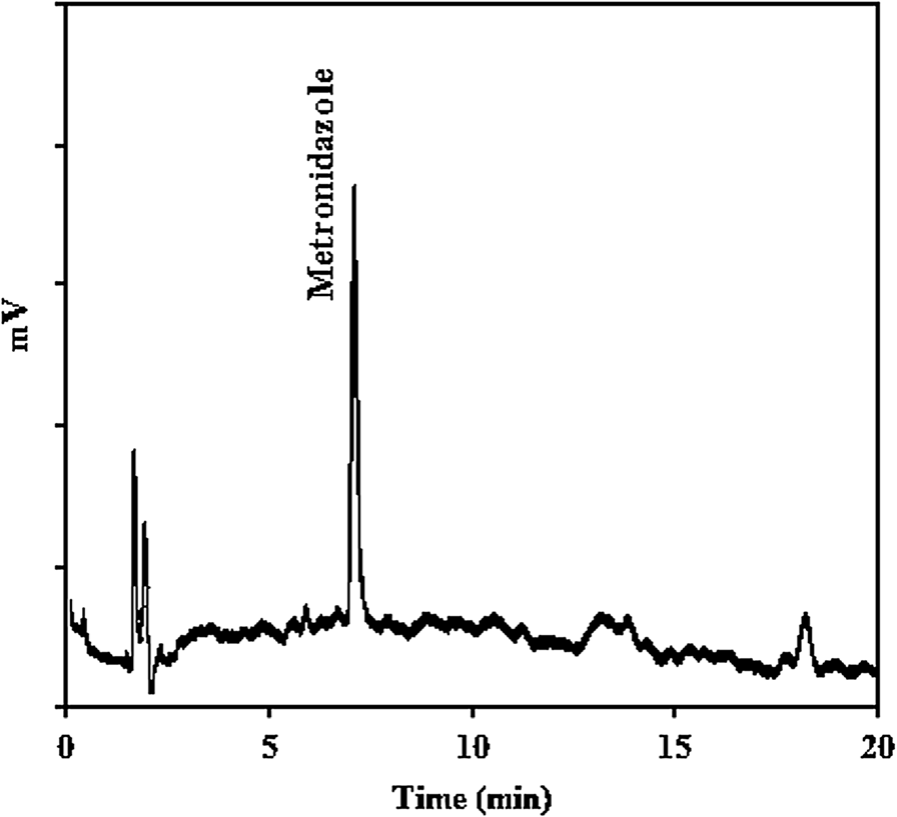
A typical HPLC chromatogram of Metronidazole.

In batch experiments, a selected dosage of TiO_2_ (0.5-3 g/L) was added in 1000 mL of MNZ solution with a certain concentration (40–120 mg/L) at different solution pH ranging from 3 to 11. Initial pH of the solution was adjusted by adding NaOH and HCl (0.1 mol/L) and measured by pH meter (Metron, Switzerland). All runs were performed under ambient conditions for 3 h. During the experiments, the solution in the photoreactor was constantly stirred and kept at constant temperature (25 ± 1°C). The MNZ solutions loaded with TiO_2_ were equilibrated in the dark for 30 min. After the equilibration period, the UV-lamp was switched on and 10 mL of the solution was taken at distinct time intervals. The aqueous samples were centrifuged (Sigma-301, Germany) at 4000 revolution per minute (rpm) for 10 min to eliminate TiO_2_ and then measured concentration of residual MNZ. The concentration of residual MNZ was determined by high performance liquid chromatography (HPLC, Waters, USA) equipped with a UV detector at 348 nm. A Diamonsil (R) C18 column (5 μm, 250 mm long × 4.6 mm ID) was used. The data were recorded by a chemistation software. The mobile phase was composed of a mixture of acetonitrile and distilled water (30/70, v/v). The flow speed was set at 1.0 mL/min and 20 μL injections were used [4]. COD was determined by COD reactor model AR851 (HACH, USA) and biodegradability was measured by five-day biochemical oxygen demand (BOD5) according to the Standard Methods [28]. A typical HPLC chromatogram of MNZ is shown in Figure 3.

The removal efficiency (%) is calculated by Eq. (1).

1 $$ \mathrm{Removal}\ \mathrm{efficiency}\ \left(\%\right) = \frac{{\mathrm{C}}_0\hbox{-} \mathrm{C}}{{\mathrm{C}}_0}\times 100 $$

where C_0_ and C are the concentrations of MNZ at initial and at time t (mg/L), respectively.

All experiments were repeated three times and the average values with error percents were reported.

## Results and discussion

### Effect of TiO_2_ dosage

**Figure 4:**
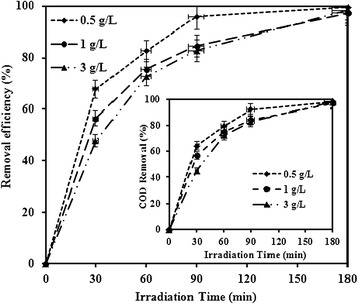
The effect of catalyst dosage on the photocatalytic degradation of MNZ and COD removal (pH = 7, MNZ =80 mg/L, BOD_5_/COD ~ 0, COD = 126 mg/L).

Effect of TiO_2_ dosage on the photocatalytic degradation of MNZ (80 mg/L) was investigated at pH 7. As shown in Figure 4, degradation efficiency of MNZ was increased from 64.28 to 97.61% by increasing irradiation time from 30 to 180 min at 0.5 g/L TiO_2_. A greater degradation efficiency of MZN was observed over the entire reaction time at low TiO_2_ dosage. This phenomena can be explained by the increased blockage of the incident UV light with increasing photocatalyst dosage [18,29]. Since the photocatalytic degradation of MNZ was not much increased over the 0.5 g/L of TiO_2_, further experiments were performed with 0.5 g/L TiO_2_. Similar results have been reported by other researchers [23,24,27].

Biodegradability of MNZ was evaluated in this work. To measure the biodegradability, BOD5 and COD values were measured before and after UV irradiation and the ratio of BOD5/COD was used as a biodegradability indicator. After 3 h reaction time, removal efficiency of COD was above 97.6% at all catalyst dosages and the ratio of BOD5/COD increased from 0 to 0.098 as the dosage increased from 0.5 to 3 g/L. This result indicates that MNZ can be changed to more biodegradable products.

### Effect of pH

**Figure 5:**
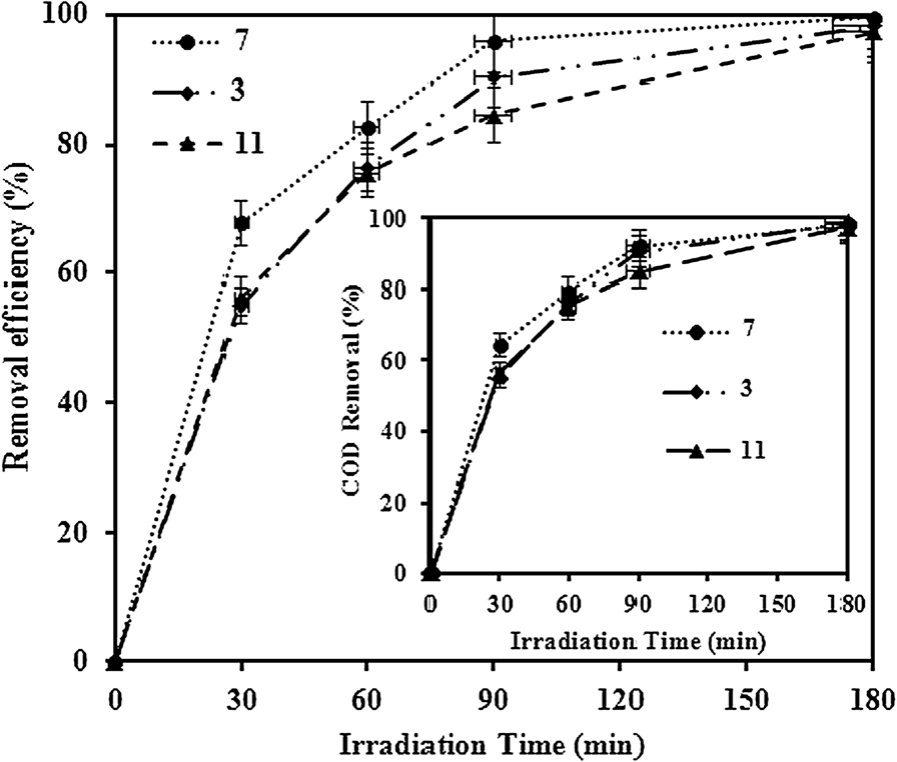
The effect of initial pH on the photocatalytic degradation of MNZ and COD removal (dosage = 0.5 g/L, MNZ =80 mg/L, BOD_5_/COD ~ 0, COD = 126 mg/L).

Effect of pH on the photocatalytic degradation of MNZ (80 mg/L) was investigated at constant TiO_2_ dosage (0.5 g/L) by varying the initial pH of solution. Figure 5 shows that the greatest degradation efficiency was obtained at neutral pH over the entire reaction time. MNZ degradation after 180 min in pH 3, 7 and 11 was 98.2, 99.48 and 97.3%, respectively. Also, COD removal after 180 min in pH 3, 7 and 11 was 97.61, 98.02 and 96.82%, respectively. This trend can be explained by the variation of charges on MNZ as well as on the surface of TiO_2_ at different solution pH. The pH_zpc_ of TiO_2_ is determined as 6.52 and pK_a_ value of MNZ is 2.55. Therefore, at acidic pH, both TiO_2_ and MNZ are positively charged, causing negative effect for the adsorption of MNZ on the surface of TiO_2_. At neutral pH, no repulsive forces between the TiO_2_ and MNZ might be developed. At basic pH, both TiO_2_ and MNZ have negative charges, causing negative effect for the adsorption of MNZ on the surface of TiO_2_. In this study, even though a distinct removal efficiency of MNZ was not observed at different solution pH, the most effective degradation of MNZ was observed at pH 7. Thus further experiments were performed at neutral pH [4,15,18].

### Effect of initial MNZ concentration

**Figure 6:**
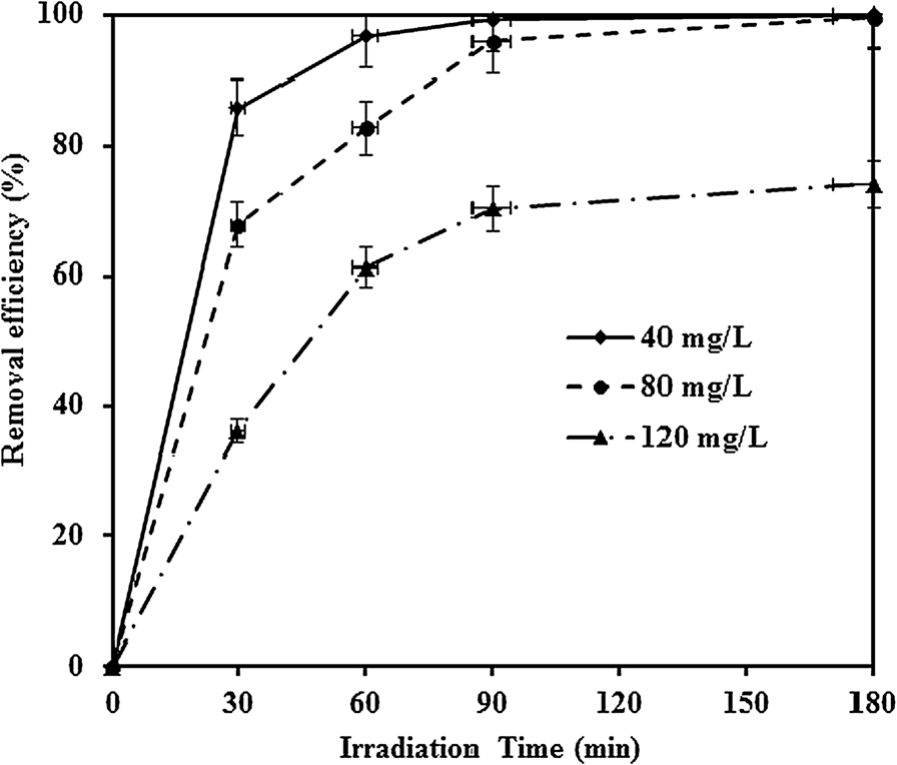
The effect of initial MNZ concentration on the photocatalytic degradation of MNZ (dosage = 0.5 g/L, pH = 7, BOD_5_/COD ~ 0, COD = 126 mg/L).

Photocatalytic degradation of MNZ by TiO_2_ was studied by varying the initial MNZ concentration at pH 7 and dosage equal to 0.5 g/L. Figure 6 shows that photocatalytic degradation efficiency decreased as the initial MNZ concentration increased. The presumed reason is that more surface of the TiO_2_ surface may be occupied by MNZ as the initial MNZ concentration increased. In addition, more degradation intermediates can be accumulated on the TiO_2_ surface, causing a negative effect in the utilization of hydroxyl radicals or positive holes in the valence band of the TiO_2_ surface. Moreover, once the concentration of the MNZ increases, more absorption of UV light by MNZ molecules, known as inner filtration effect, can occur. This effect causes decrease of photons reaching to the TiO_2_ surface [4]. Similar results have been reported by other researchers [15,18,23].

### Kinetic study and electrical energy determination

In order to obtain kinetic information, experimental result in Figure 6 was fitted with a pseudo-first-order equation as expressed in Eq. (2).

2 $$ \ln \left[{\left(\mathrm{M}\mathrm{N}\mathrm{Z}\right)}_0/{\left(\mathrm{M}\mathrm{N}\mathrm{Z}\right)}_{\mathrm{t}}\right]={\mathrm{k}}_1\mathrm{t} $$

**Figure 7:**
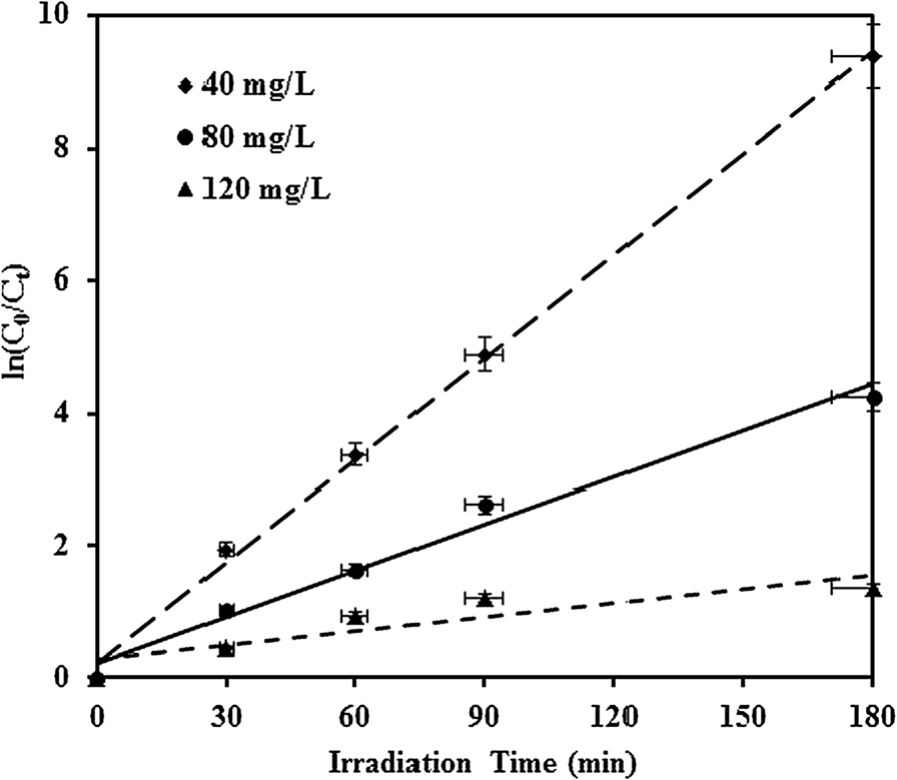
First-order kinetic model for the photocatalytic degradation of MNZ (dosage = 0.5 g/L, pH = 7, BOD_5_/COD ~ 0, COD = 126 mg/L).

To calculate the rate constant from the plot ln[C_0_/C] versus t, only initial data points were considered. Figure 7 shows the plot of ln[C_0_/C] versus t for the degradation of MNZ. The first-order rate constants of photocatalytic process (k_obs_ (1/min)) at different initial concentrations of MNZ are summarized in Table 2. The relationship between the initial photocatalytic degradation rate (r) and the initial concentration of organic substrate for a heterogeneous photocatalytic process can be described by Langmuir–Hinshelwood (L-H) model (Eqs. 3 and 4) [30,31]:

**Table 2:** **Pseudo-first order kinetic parameters and E**_**Eo**_ **values for the photocatalytic degradation of MNZ at different initial MNZ concentrations (catalyst dose = 0.5 g/L and pH =7)**

**E**_**Eo**_ **(kWh/m** ^**3**^ **)**	**R** ^**2**^	**1/k**_**obs**_ **(min)**	**k**_**obs**_ **(1/min)**	**[MNZ]**_**0**_ **(mg/L)**
93.57	0.998	19.493	0.0513	40
206.01	0.9828	42.918	0.0233	80
666.67	0.7874	138.888	0.0072	120

3 $$ \mathrm{r}=\frac{{\mathrm{k}}_{\mathrm{c}}{\mathrm{K}}_{\mathrm{MNZ}}\ \left[\mathrm{M}\mathrm{N}\mathrm{Z}\right]}{1+{\mathrm{K}}_{\mathrm{MNZ}}{\left[\mathrm{M}\mathrm{N}\mathrm{Z}\right]}_0}={\mathrm{k}}_{\mathrm{obs}}\left[\mathrm{M}\mathrm{N}\mathrm{Z}\right] $$

4 $$ \frac{1}{{\mathrm{k}}_{\mathrm{obs}}}=\frac{1}{{\mathrm{k}}_{\mathrm{c}}\ {\mathrm{K}}_{\mathrm{MNZ}}}+\frac{{\left[\mathrm{M}\mathrm{N}\mathrm{Z}\right]}_0}{{\mathrm{k}}_{\mathrm{c}}} $$

where [MNZ]_0_ is the initial concentration of the antibiotic in mg/L, k_c_ (mg/L/min) is the kinetic rate constant of surface reaction and K_MNZ_ (L/mg) is the Langmuir adsorption constant. The values of K_MNZ_ and k_c_ were obtained as 0.0285 L/mg and 0.67 mg/L/min, respectively. This L–H kinetic model has been used by several authors to analyze heterogeneous photocatalytic reactions [30,31].

For the case of photocatalytic reaction, electrical energy is very important factor for the real application and evaluation for the electrical energy should be provided. Thus, in this work, electrical energy was evaluated by calculating electrical energy per order (E_EO_). It is defined as the number of kWh of electrical energy required to reduce concentration of a pollutant by 1 order of magnitude (90%) in 1 m^3^ of contaminated water. The E_EO_ (kWh/m^3^) can be calculated from the following equation:

5 $$ {E}_{EO}=\frac{\mathrm{p}\times \mathrm{t}\times 1000}{\mathrm{V}\times 60\times \log \left({\mathrm{C}}_1/{\mathrm{C}}_{\mathrm{f}}\right)} $$

6 $$ {E}_{EO}=\frac{38.4 \times \mathrm{P}}{{\mathrm{V} \times \mathrm{k}}_{obs}} $$

where P is the rated power (kW) of the AOP system, t is the irradiation time (min), *k*_*obs*_ is the pseudo-first order rate constant (1/min), V is the volume (L) of the wastewater in the reactor, C_i_ and C_f_ is the initial and final MNZ concentrations, respectively. The E_EO_ value for UV-alone and UV/TiO_2_ processes are reported in Table 3. E_EO_ value for UV/TiO_2_ process was lower than UV-alone process.

**Table 3:** **The E**_**Eo**_ **values for the removal of MNZ ([MNZ]**_**0**_ **= 80 mg/L, catalyst dose = 0.5 g/L and pH =7)**

**Process**	**E**_**Eo**_ **(kWh/m** ^**3**^ **)**
UV 8 W-alone	290.11
UV125W-alone	245.17
UV 8 W/TiO_2_	230.04
UV 125 W/TiO_2_	197.95

### Comparison of different MNZ removal processes and reusability test

**Figure 8:**
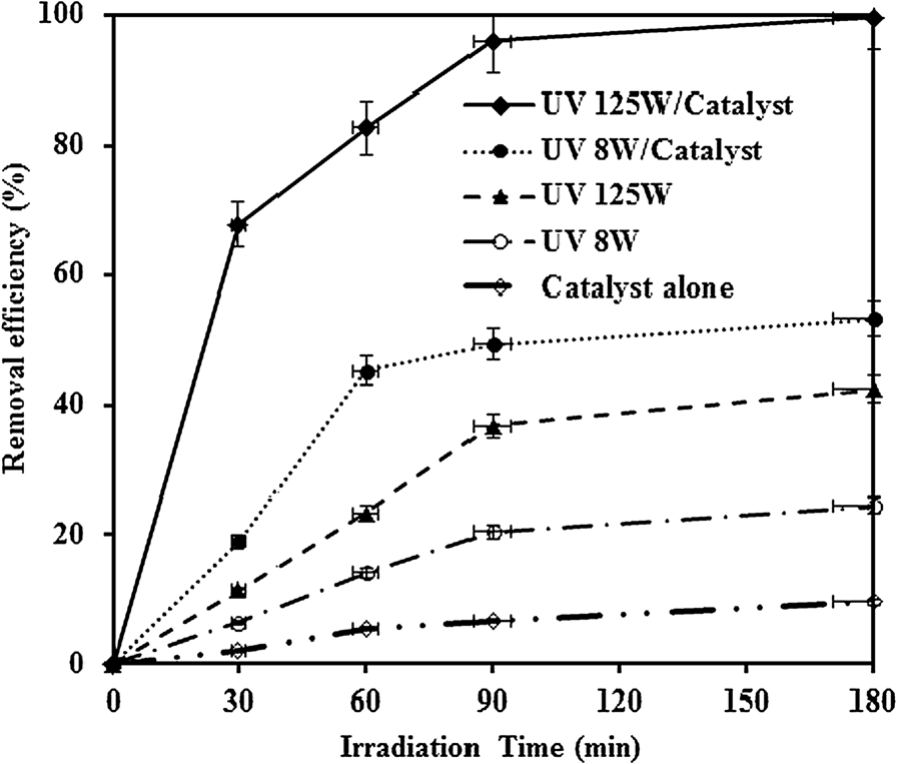
The contribution of each process involved in the photocatalytic degradation of MNZ (dosage = 0.5 g/L, pH = 7, MNZ = 80 mg/L, BOD_5_/COD ~ 0, COD = 126 mg/L).

MNZ removal by TiO_2_-alone, UV 8 W-alone, UV 125 W-alone, UV 8 W/TiO_2_ and UV 125 W/TiO_2_ processes are shown in Figure 8. Removal efficiency of MNZ through adsorption process (TiO_2_-alone) was low. Overall removal efficiency of MNZ by TiO_2_-alone, UV 8 W-alone, UV 125 W-alone and UV 8 W/TiO_2_ process was 9.63%, 24.52, 42.32% and 53.53%, respectively. But 99.48% of MNZ was removed with UV 125 W/TiO_2_. These experiments demonstrate that both UV light and TiO_2_ are necessary for the effective degradation of MNZ.

Based on the above experiments and analysis, mechanism of the photocatalysis could be proposed as following:

An electron excites from the valence band to the conduction band of TiO_2_, generating electron–hole pair, with UV light (λ < 390 nm) (Eq. 7) [24,27]:

7 $$ \mathrm{T}\mathrm{i}\mathrm{O}2 + \mathrm{h}\upupsilon\ \to \mathrm{T}\mathrm{i}\mathrm{O}2\left({\mathrm{e}}_{\mathrm{CB}}^{\hbox{-} }+{\mathrm{h}}_{\mathrm{VB}}^{+}\right) $$

Then, the generated electron–hole pairs can participate in the reactions with electron acceptors like O_2_ and donors like H_2_O or OH^−^ to generate highly reactive radical species particularly hydroxyl radicals (E^0^ = +3.06 V), which can oxidize organic contaminants and their degradation intermediates unselectively. Furthermore, positive holes can oxidize pollutants directly, too (Eqs. 8–11) [24,27]. Also Homem and Santos [32] reviewed degradation and removal methods of antibiotics from aqueous matrices and suggested removal mechanisms.

8 $$ {\mathrm{h}}_{\mathrm{VB}}^{+}+\mathrm{M}\mathrm{N}\mathrm{Z}\ \to \mathrm{intermediates}\ \mathrm{or}\ \mathrm{products} $$

9 $$ {\mathrm{h}}_{\mathrm{VB}}^{+}+{\mathrm{H}}_2\mathrm{O}\ \to {\mathrm{H}}^{+}+\cdotp \cdot \mathrm{O}\mathrm{H} $$

10 $$ {\mathrm{h}}_{\mathrm{VB}}^{+} + {\mathrm{OH}}^{-}\to \cdotp \cdotp \cdot \mathrm{O}\mathrm{H} $$

11 $$ \cdotp \cdot \mathrm{O}\mathrm{H}+\mathrm{M}\mathrm{N}\mathrm{Z}\to \kern0.5em \mathrm{intermediates}\kern0.5em \mathrm{or}\kern0.5em \mathrm{products} $$

**Figure 9:**
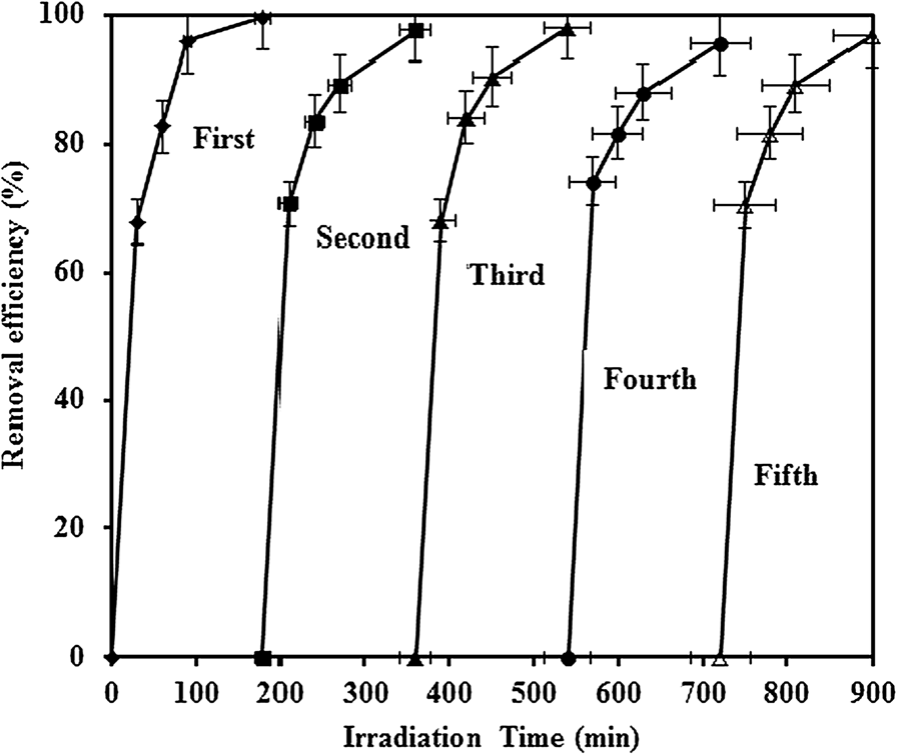
Reusability test of UV/TiO_2_ for the photocatalytic degradation of MNZ over five consecutive runs (dosage = 0.5 g/L, pH = 7, MNZ =80 mg/L, BOD_5_/COD ~ 0, COD = 126 mg/L).

The reusability of a photocatalyst is an important factor for real application. Hence, five consecutive photocatalytic experiments were performed by UV/TiO_2_ process. As can be seen in Figure 9, quite similar photocatalytic activity was maintained over five consecutive runs. Photocatalytic degradation of MNZ with illuminated TiO_2_ was compared with other reported data. Removal efficiency and reaction rate constant were compared and summarized in Table 4.

**Table 4:** **Comparison of photocatalytic degradation of MNZ**

**Systems**	**pH**	**Catalyst dosage (g/L)**	**[MNZ** **]**_**0 **_ **(mg/L)**	**Lamp (W)**	**Time (min)**	**Removal efficiency (%)**	**k**_**obs**_ **(min** ^**−1**^ **)**	**Reference**
Visible/ZnO	-	1.0	5	300	160	16.9	-	[3]
Visible/ZnO-RGO	-	1.0	5	300	160	49.3	-	[3]
Visible	-	-	5	300	160	5.5	-	[3]
UV/ZnO	10	1.5	80	125	180	96.55	-	[4]
UV	6	-	0.006	Lp Mp	5 5	6 12	0.005616 0.02304	[16]
UV/Niobate K_6_Nb_10.8_O_30_	-	1.5	10	18	180	57	0.00449	[18]
UV/TiO_2_	7	0.5	80	125	120	99.48	0.0233	Present study

## Conclusions

From the application of TiO_2_ for the photocatalytic degradation of MNZ in aqueous solutions, a maximum removal of MNZ was observed at neutral pH. Removal efficiency was decreased by increasing TiO_2_ dosage and initial MNZ concentration. Electrical energy per order was increased and reaction rate constant was decreased with increasing initial MNZ concentration. Photocatalytic activity was maintained even after five consecutive runs. Finally, UV/TiO_2_ is identified as a promising technique for the removal of MNZ with high efficiency in a relatively short reaction time.
